# Several inaccurate or erroneous conceptions and misleading propaganda about brain-computer interfaces

**DOI:** 10.3389/fnhum.2024.1391550

**Published:** 2024-03-27

**Authors:** Yanxiao Chen, Fan Wang, Tianwen Li, Lei Zhao, Anmin Gong, Wenya Nan, Peng Ding, Yunfa Fu

**Affiliations:** ^1^Faculty of Information Engineering and Automation, Kunming University of Science and Technology, Kunming, China; ^2^Brain Cognition and Brain-Computer Intelligence Integration Group, Kunming University of Science and Technology, Kunming, China; ^3^Faculty of Science, Kunming University of Science and Technology, Kunming, China; ^4^School of Information Engineering, Chinese People’s Armed Police Force Engineering University, Xi’an, China; ^5^Department of Psychology, School of Education, Shanghai Normal University, Shanghai, China

**Keywords:** misleading propaganda about BCI, mind reading, mind-controlled, controlling brain, “intelligence” of BCI

## Abstract

Brain-computer interface (BCI) is a revolutionizing human-computer interaction, which has potential applications for specific individuals or groups in specific scenarios. Extensive research has been conducted on the principles and implementation methods of BCI, and efforts are currently being made to bridge the gap from research to real-world applications. However, there are inaccurate or erroneous conceptions about BCI among some members of the public, and certain media outlets, as well as some BCI researchers, developers, manufacturers, and regulators, propagate misleading or overhyped claims about BCI technology. Therefore, this article summarizes the several misconceptions and misleading propaganda about BCI, including BCI being capable of “mind-controlled,” “controlling brain,” “mind reading,” and the ability to “download” or “upload” information from or to the brain using BCI, among others. Finally, the limitations (shortcomings) and limits (boundaries) of BCI, as well as the necessity of conducting research aimed at countering BCI systems are discussed, and several suggestions are offered to reduce misconceptions and misleading claims about BCI.

## 1 Introduction

Brain-computer interface (BCI) is a revolutionizing human-computer interaction that establishes a new channel of communication and control directly between brain and external devices, bypassing peripheral nerves and muscles (Graimann et al., 2010). Studies have demonstrated the efficacy of BCI in monitoring, replacing, improving/restoring, enhancing, and supplementing impaired or injured natural central nervous system outputs and inputs, indicating significant potential applications in the medical and health fields (Wolpaw, 2013; Ramsey and Millán, 2020). With the development of BCI technology, its integration with advanced artificial intelligence could bring profound changes to medical and health practices (Miller and Brown, 2018).

Although BCI, which aims to be widely applied, still has a long way to go, BCI products for specific individuals or groups in specific scenarios are expected to emerge in the near future. Extensive research has been conducted on the principles and implementation methods of BCI (Wolpaw, 2013; Ramsey and Millán, 2020), and efforts are currently focused on bridging the gap from research to real-world applications (Allison et al., 2012). However, some members of the public have inaccurate or erroneous perceptions of BCI. Additionally, certain media outlets, as well as some BCI researchers, developers, manufacturers, or regulators of BCI technology, engage in misleading or sensationalized propaganda about BCI, which leads to excessively high expectations among the public.

Therefore, this paper summarizes the several inaccurate or erroneous cognitions and misleading propaganda about BCI. These include claims that BCI has widespread applications, significant efficacy, a high level of maturity, a large market size, “intelligence,” and the ability to “download” a person’s mind or memories into a computer, as well as the capability to “upload” or “write” information into the brain. They also cover beliefs that BCI enables “mind-controlled,” “controlling brain,” “mind reading,” automatic recognition of users’ intentions, and significantly improved memory, cognition, and behavioral performance.

Furthermore, they include the notion that users are not an integral part of the BCI system, the belief that ethics are not included in the standards for BCI technology, and the idea that technologies like neural stimulation and neurofeedback are not encompassed within the scope of BCI. Additionally, there is a belief that non-invasive BCIs pose no ethical risks, and concerns about neural privacy issues brought by BCI are often exaggerated.

Finally, the limitations (shortcomings or weaknesses) and limits (maximum potential or boundaries) of BCI technology are discussed in the paper. What efficacies can the current BCIs provide? What level of performance have they achieved? To what extent have they been developed? What can they not do? What is the current status of the industrial translation of BCIs? What are the pathways for BCIs to be translated into practical applications? Regarding the potential applications of BCI, which could be realized in the near future, which within the next 5 years, which within the next 10 years, and which are still dream or imagined situations? Is it necessary to conduct “research on countering BCI systems”? Several suggestions are offered to reduce misconceptions and misleading claims about BCI in the paper.

This paper recommends a scientific, objective, and rational approach to BCI technology. It advises that research reporting, industry dissemination, and media coverage should eschew sensationalizing BCI technology for profit motives, aiming to prevent the formation of a BCI industry or economic bubble. The objective is to diminish the spread of inaccurate or erroneous cognitions and misleading propaganda about BCI.

## 2 Several inaccurate or erroneous conceptions and misleading propaganda about BCI

During the development and promotion of BCI technology, certain inaccurate or erroneous conceptions, along with misleading propaganda, have the potential to distort the public’s genuine understanding of the technology. The general public’s understanding of BCI technology mostly comes from media reports, and some media may exaggerate facts to attract viewers. Some BCI developers and manufacturers might over-promote their products to attract investment or consumers. The general public lacks a deep understanding of the complexity and difficulty of BCI technology, as well as the current level of the technological advancement.

### 2.1 BCI has been widely used

Contrary to some academic reports and media assertions, BCI has not yet achieved widespread use. This representation greatly diverges from the current reality of BCI applications. At present, BCI technology is predominantly still in the stages of laboratory research and clinical trials, without having been adopted on a large scale. Even in hospital departments where BCI has potential applications, it is seldom seen in practical use, let alone being prevalent in everyday life (Aricò et al., 2018). While neuromodulation devices such as transcranial stimulation or deep brain stimulation can be included under the BCI umbrella, their applications are currently limited to certain hospital settings (Elias et al., 2018; Ajrawi et al., 2021; Elyamany et al., 2021; Zhao et al., 2023). Before BCIs can achieve either small or large-scale widespread use, several critical scientific and technical challenges must be overcome (Ramsey and Millán, 2020; Yadav et al., 2020; Li et al., 2021; Aggarwal and Chugh, 2022).

Practical BCIs need to bridge the gap between research and real-world applications (Allison et al., 2012). Currently, the usability and user satisfaction of existing BCI systems are low, and their medical efficacy (such as the efficacy of motor imagery BCIs in active rehabilitation) requires rigorous evidence-based research and objective evaluation (Baniqued et al., 2021; Kim et al., 2021; Mridha et al., 2021). The irreplaceability of BCIs, characterized by the difficulty of existing technologies to substitute or surpass them, along with their accessibility and user acceptability, still requires significant enhancements (Morone et al., 2015; Bernal G. et al., 2021; Williams et al., 2022).

The correct understanding should be that BCI is a revolutionary human-computer interaction technology with significant potential applications (Graimann et al., 2010). However, exaggerating these potential applications can lead to unrealistic expectations. Although BCIs are currently not widely used and may not achieve widespread use in the future, they hold significant potential for specific applications in certain groups or individuals. These include those with Amyotrophic Lateral Sclerosis (ALS), traumatic brain injury, spinal cord injury, locked-in syndrome, severe brain damage with consciousness disorders, Parkinson’s disease, Alzheimer’s disease, severe emotional disorders, and motor disorders induced by stroke, such as impairments in hand and lower limb mobility (Ramsey and Millán, 2020). For BCIs to be effectively applied in everyday life, they need to meet the actual needs of users and enhance their experience.

### 2.2 BCI technology has significant efficacy

Some academic reports and media promotions have exaggerated the efficacy of BCI technology, potentially leading to excessively high public expectations. Although existing literature suggests that BCI technology has capabilities for monitoring, replacing, improving/restoring, enhancing, and supplementing functions, the magnitude and degree of these effects still require objective evaluation (Kothe and Makeig, 2013; Ramsey and Millán, 2020; Gu et al., 2021; Moses et al., 2021; Xu et al., 2021; Rouzitalab et al., 2023). For example, the methods for evaluating the effectiveness of BCI in the treatment or rehabilitation of central nervous system-related diseases/disorders remain unclear or non-standardized. This calls for collaboration among researchers in BCI clinical translation, manufacturers, clinicians, and patients, to objectively assess the medical efficacy of BCI. It is crucial to avoid subjective evaluations or the creation of hype for profit (Morone et al., 2015).

In clinical practice, the efficacy of medications or treatment methods is typically established through randomized double-blind controlled trials, and this standard approach is equally applicable to the verification of BCI efficacy. Moreover, if BCI is proven to be effective, it is essential to elucidate the underlying mechanisms of its therapeutic action. Clinical scales pertinent to central nervous system diseases and disorders encompass both objective indicators, as measured by medical instruments (such as assessments of muscle strength and electromyography results), and scales that evaluate the extent of improvement in clinical symptoms.

### 2.3 The maturity level of BCI technology is relatively high

Some academic reports and media outlets highlight the advancements and advantages of BCI technology, frequently neglecting to mention its limitations. This portrayal, whether intentional or not, can imply a high level of maturity in BCI technology, potentially leading to skewed perceptions of its technical capabilities among the general public. However, BCI technology is still in its infancy (Ramsey, 2020). The grand challenges are in front of us, and the breakthrough of BCI technologies requires the collaborative efforts of scientists from multiple disciplines (Bergeron et al., 2023). The research and development of BCI technology require interdisciplinary knowledge and technologies, including neuroscience, man–machine engineering or ergonomics, signal processing, machine learning, materials science and so on. BCI technology needs to integrate these fields, hence its development is complex, and the current level of the technological maturity and practicality needs significant improvement.

It is particularly noteworthy that BCI technology is closely related to man–machine engineering or ergonomics, facing challenges in coordination among the human brain (as a biological adaptive controller), BCI adaptive algorithms, and machines (Taylor et al., 2002; Wolpaw et al., 2002; McFarland et al., 2012). The research methods and evaluation metrics of BCI involve multiple disciplines such as psychology, physiology, medicine, anthropometry, esthetics, design, and engineering technology, aiming to enhance the characteristics of BCI in terms of efficiency, safety, and comfort.

Currently, human understanding of the brain is limited, and the development of BCI technology is constrained by the understanding of brain functions and neural coding methods. Moreover, the ethical and safety issues involved in BCI also limit its research and development.

While BCI technology has achieved commendable progress, there is a pressing need for breakthroughs in brain signal acquisition. Currently, the overall user satisfaction with BCI sensors, including aspects such as safety, comfort, ease of use, and esthetic design, remains relatively low. This is particularly true in terms of the safety and long-term stability of implantable BCI systems. Moreover, current BCI paradigms are significantly limited and call for a transformative breakthrough beyond the traditional, classic paradigms like SSVEP-BCI, P300-BCI, and MI-BCI. There’s a compelling need to innovate and develop more intuitive and effective interactive BCI paradigms (Willett et al., 2021, 2023; Metzger et al., 2023).

### 2.4 The market size of BCI is very large

As mentioned in Section 2.3, the BCI field is still in its infancy, the market size is unknown, which places high commercial risks with potential manufacturers. As a result, such devices are not yet available (Ramsey, 2020). However, some players in the BCI industry, including manufacturers, certain media outlets, and even some researchers, promote the idea that the BCI market has a significant size. Yet, their assessments of the potential market capacity tend to be subjective (Yin et al., 2023).

The actual efficacy of several BCI products available in the market is challenging to quantify (Kawala-Sterniuk et al., 2021), and some of these products have not demonstrated any efficacy. The post-purchase usage of BCI products is not promising, with minimal usage leading to little or no improvement in users’ quality of life. Additionally, there are concerns about the presence of inferior or counterfeit BCI products in the market. Surveys among potential end-users with medical conditions, professionals in Assistive Technology (AT), and AT distributors indicate that the currently available BCI products in the market do not align with the design requirements of the end-users (Gao et al., 2021).

**Figure 1:**
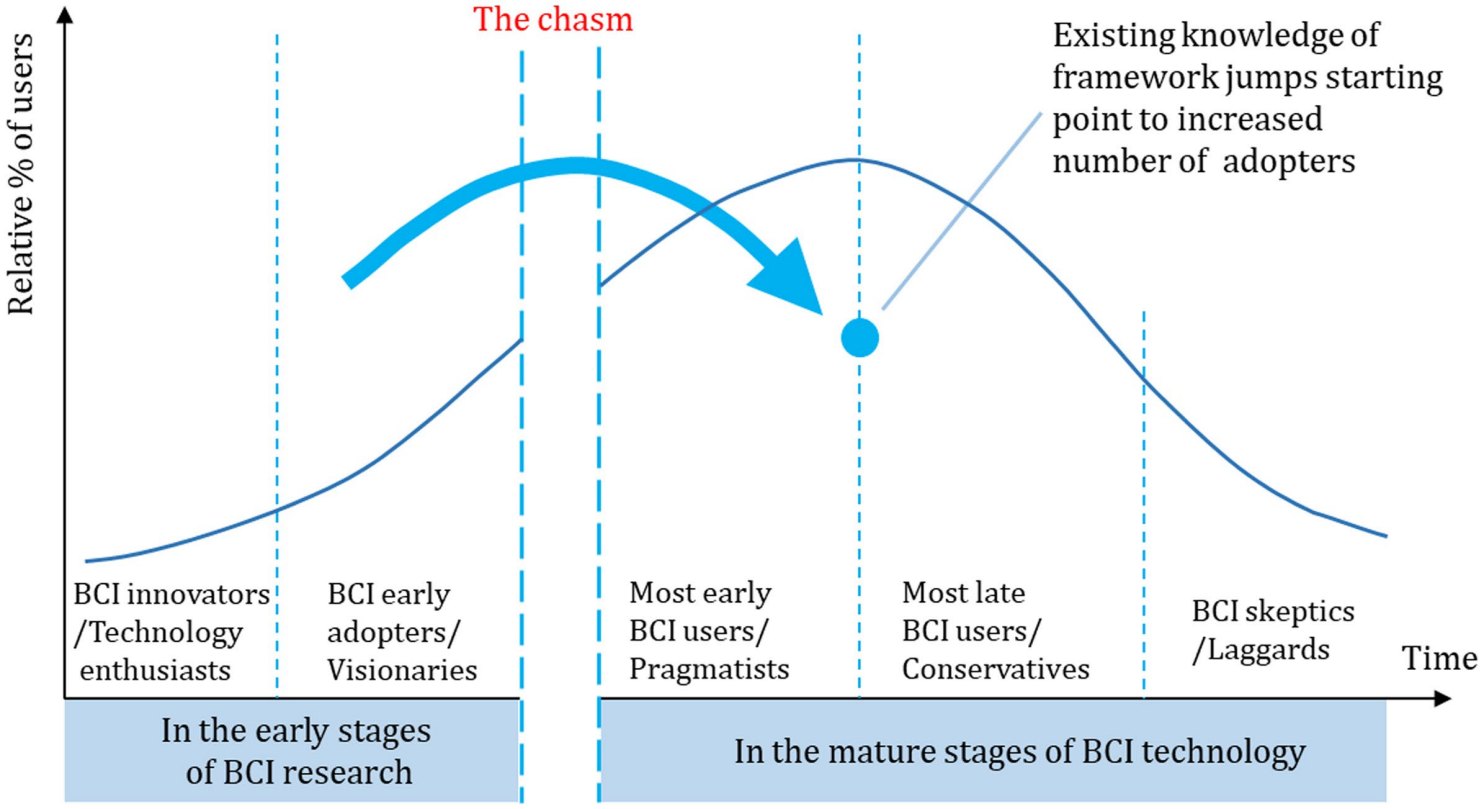
Process for translating BCI into the real-world applications referencing the technology adoption lifecycle (TALC) (Pulliam et al., 2020; Luo et al., 2022).

Currently, the development of BCI technology still confronts a significant translational gap: there is insufficient knowledge on how to transition BCIs from the laboratory to practical settings, and BCI-controlled applications lack in terms of usability and accessibility (Kapgate, 2022). Addressing these issues of usability and accessibility is crucial in the development of BCI technology to overcome this translational challenge. Figure 1 demonstrates the process of BCI’s translation into practical applications, referencing the Technology Adoption Lifecycle (TALC) (Pulliam et al., 2020; Luo et al., 2022).

### 2.5 The “intelligence” of BCI

Intelligence encompasses abilities such as abstraction, logic, understanding, self-awareness, learning, emotional knowledge, reasoning, planning, creativity, critical thinking, and problem-solving. Additionally, it can be characterized as the capacity to perceive or infer information, which is then retained as knowledge and applied in adaptive behavior within various environments or contexts (Obaid, 2023). Beyond biological intelligence, which pertains to humans, animals, and plants, intelligence manifested in computers or other machines is referred to as artificial intelligence (Natale and Ballatore, 2020). Some researchers posit that BCI technology has the potential to serve as a bridge between biological and artificial intelligence. However, concrete plans on how to accomplish this have yet to be outlined. What comprises the intelligence inherent in BCI itself, and at what level does this intelligence operate?

Typically, BCI technology includes BCI paradigms, as well as neural encoding and decoding processes. The BCI paradigm consists of a set of carefully designed external stimuli or mental activities created in advance by developers. The BCI is only capable of decoding user intentions specified by the paradigm with a certain level of accuracy (Tai et al., 2024), and its intelligence level is currently quite limited. Nevertheless, some BCI researchers, manufacturers, and media outlets portray BCI as highly intelligent, often overstating the technology with terms like “intelligence” or “intelligent” BCI. Such promotions are not in line with the actual capabilities of BCI and risk leading the public to form overly high expectations of its intelligence. It’s crucial to acknowledge the limitations in the intelligence aspect of BCI, which include theoretical deficiencies, technical constraints, application field limitations, and challenges in interdisciplinary integration. At the current stage, overemphasizing the “intelligence” of BCI or labeling it as “intelligent” is not appropriate. One potential strategy to enhance the intelligence level of BCI technology is integrating it with advanced AI systems.

### 2.6 “Mind-controlled” by BCI

Some BCI researchers, developers, manufacturers, and media outlets refer to BCI-based brain-controlled technology as “mind-controlled.” This term implies that users can control complex external devices solely through their consciousness or will. Although BCI is capable of translating specific brain activities of users into control signals with a certain level of accuracy and speed, the extent of this control is often quite limited. Users require extensive training and adaptation (Benaroch et al., 2021; Kapgate, 2022) and must adhere to the pre-established BCI paradigm (Tai et al., 2024). If not, the concept of “mind-controlled” becomes ineffective. The “thought-controlled” external devices or computers reported in the media are all operated by mental activities or external stimuli, as defined by the BCI paradigm (Tai et al., 2024).

The term “mind-controlled” BCI may exaggerate the capabilities of brain-controlled technology, potentially leading some of the public to associate it with parapsychological phenomena such as bending spoons and telekinesis (Kripal, 2015). To prevent setting unrealistic expectations about brain-controlled technology based on BCI, it’s recommended to avoid terms such as “mind-controlled” or “thought-controlled,” and to inform the public about the technology’s limitations.

### 2.7 “Controlling brain” by BCI

Some researchers, developers, and manufacturers in the field of BCI, as well as certain media outlets, use the term “controlling brain” when discussing BCI-based neural stimulation and neurofeedback techniques. They promote the idea that BCI is capable of directing animal movements based on human intentions and claim that scientists are in the process of developing “controlling brain” technologies using BCI. Such terminology and promotional tactics can lead to public concerns and misunderstandings, raising questions about the objectives of BCI research. This includes potential negative impacts on the physical and mental health of humans or animals, as well as significant ethical issues, such as violations of ethical standards or the potential triggering of mental disorders. For example, there have been claims such as “My brain has been controlled by an implantable BCI chip; I am a victim of controlling brain technology. “Who would want their brain to be controlled? To prevent public misconceptions regarding BCI-based neural stimulation and neurofeedback technologies, it is discouraged to use terms such as “controlling brain.”

To date, BCI-based neural stimulation technology has primarily been used in experiments involving animals and human subjects. Bonkon Koo and his colleagues have utilized a brain-to-brain interface (BBI) system, in which the visual evoked potentials of human subjects are used to stimulate the nigrostriatal pathway in rats, thereby controlling their movements (Koo et al., 2017). The goal is to improve the interaction between humans and untrained animals, contributing to a better understanding of animal behavior. All experimental procedures were ethically approved by the Institutional Review Boards and Animal Care and Use Committees of Hanlim University in Korea. Cheol-Hu Kim and his colleagues have utilized a BCI protocol that combines event-related desynchronization (ERD) with steady-state visual evoked potentials (SSVEP), using a stimulation device specifically designed for turtles. This device triggers their instinctive escape behavior, allowing for remote control of their movement paths in both indoor and outdoor settings (Kim et al., 2016). The aim of this study is to develop a framework for future interactions between human subjects and animals. It has received approval from the Institutional Review Boards and Animal Care and Use Committees of KAIST in Korea. While these studies focus on influencing the animals’ navigational capabilities rather than exerting arbitrary control, such research still faces resistance from some segments of the public, despite obtaining ethical approvals.

**Figure 2:**
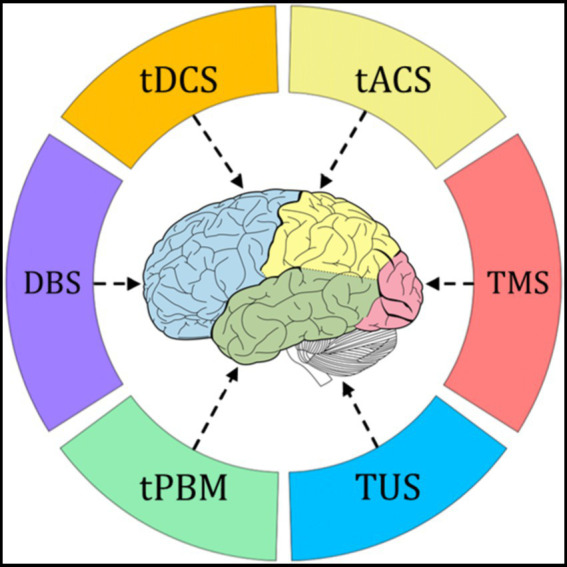
A schematic diagram of BCI based neural stimulation technology, in which external devices input electrical, magnetic, acoustic, and optical nerve stimuli to the brain (Morone et al., 2015).

Research and applications of BCI-based neural stimulation in human subjects or users encompass a variety of techniques, including transcranial electrical stimulation, transcranial magnetic stimulation, transcranial ultrasound stimulation, transcranial photobiomodulation (tPBM), implanted electrical stimulation (such as deep brain stimulation), and multimodal bidirectional closed-loop brain stimulation, as illustrated in Figure 2. These neural stimulation technologies are primarily used to treat a range of neurological conditions, such as movement disorders, dementia, cognitive impairments, attention deficits, autism spectrum disorders, depression, bipolar disorders, anxiety disorders, schizophrenia, obsessive-compulsive disorder, addiction, sleep disorders, pain disorders, epilepsy, stroke, and consciousness disorders. They aim to facilitate the rehabilitation of these conditions (Philip et al., 2017). All related studies and applications have been approved by the relevant ethical and moral committees.

BCI-based neurofeedback (NF) is a form of biofeedback training that uses electroencephalography (EEG), or “brain waves, “as the signal for controlling feedback. EEG sensors are placed on the subject’s scalp to capture brain signals, and a computer and software interface convert these signals into feedback through human-machine interaction. Neurofeedback uses visual, auditory (sound), or tactile feedback to facilitate a learning process in the brain. The main objective of this technique is to promote relaxation of the brain by increasing alpha waves or related rhythms. Additionally, it can provide various other benefits by enhancing the capacity of the Central Nervous System (CNS) to regulate cycles of attention and relaxation, as well as improving brain connectivity (Wolpaw, 2013). Neurofeedback training is considered safe, and studies or applications involving human subjects have received approval from relevant ethical and moral committees.

BCI technology, which transmits commands from the brain, primarily focuses on directly controlling external devices, such as wheelchairs or computer cursors operated via brain commands, rather than exerting direct control over the brain itself (Palumbo et al., 2021). However, certain misleading promotions have overstated the capabilities of BCI, labeling it as a “controlling brain” technology. In reality, the current state of BCI technology does not allow for arbitrary control over brain functions. Ethical committees should not approve research into “controlling brain” technologies that fail to enhance human quality of life, and such studies should be prohibited by law and regulation. Likewise, the term “emotion manipulation” may mislead the public into believing that BCI has the capability to control or change an individual’s emotional states (Bernal S. L. et al., 2021). However, this technology is still at an early stage, and its “controlling brain” capabilities should not be exaggerated.

### 2.8 “Mind reading” by BCI

Some BCI researchers and developers, particularly media outlets, claim that BCI can “read” users’ thoughts, referring to it as “mind reading” with BCI. Such publicity is inaccurate and may lead the public to erroneously believe that BCI can access any of a person’s thoughts. The concept of BCI “mind reading” could provoke concern or fear among the public. For instance, concerns may arise regarding the potential invasion of personal privacy through mind reading, which could result in skepticism about the goals of BCI research and overall dissatisfaction with the field.

**Figure 3:**
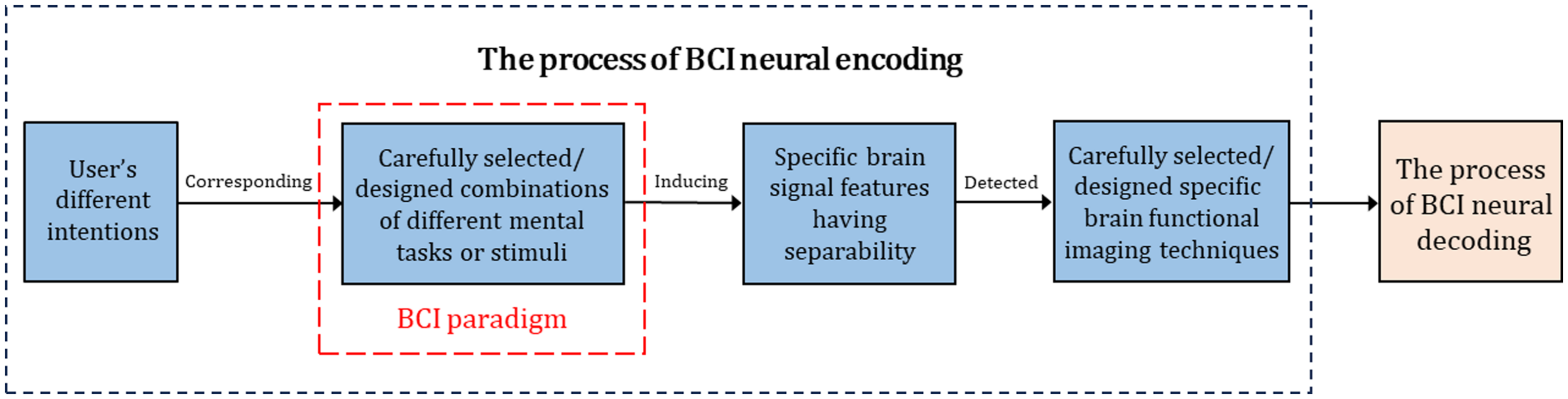
BCI identifies the user’s intentions based on a set of external stimuli or mental tasks (BCI paradigms) carefully designed or selected in advance by researchers and developers (Tai et al., 2024).

BCI operates by identifying a user’s intentions through a predefined set of external stimuli or mental tasks, known as BCI paradigms, which are carefully designed or selected by researchers and developers (as shown in Figure 3). Moreover, BCI can only achieve encoding and decoding with a certain level of accuracy and is challenged to consistently reach 100% accuracy (Tai et al., 2024). This implies that if users do not engage with the specified external stimuli or perform the designated mental tasks, the BCI system will struggle to accurately discern their intentions (Maiseli et al., 2023). Therefore, BCI is incapable of reading an individual’s random thoughts. Our current knowledge of the brain’s structure and function remains quite limited, especially regarding how information is stored and processed within it. Moreover, existing brain imaging technologies have limitations and are not equipped to intricately interpret complex brain data, such as memories and thoughts. To avoid misleading the public, it is recommended to refrain from using the term “mind reading” in relation to BCI.

### 2.9 BCI can “automatically” recognize the user’s intentions

Some BCI researchers, developers, manufacturers, and media outlets claim that BCI can “automatically” recognize users’ intentions. This claim might mislead the public into believing that BCI is a highly automated system capable of identifying any user intention. However, BCI is more accurately described as a semi-automated system with “human-in-the-loop” (or “brain-in-the-loop”) dynamics (Lyu et al., 2023), which relies on user engagement and collaboration under specific conditions (Roc et al., 2021). Automated systems, also known as unmanned systems, operate without human involvement. As illustrated in Figure 3, both the BCI user and their brain are essential components of the entire online BCI system, playing a critical role in its effective functioning. Additionally, as outlined in Section 2.8, BCI is unable to recognize random intentions of the users; it can only decode, with a certain level of accuracy, the external stimuli or mental tasks defined within the BCI paradigms.

### 2.10 “Downloading” an individual’s thoughts or memories into a computer using BCI

Some media outlets claim that BCI technology could potentially “download” an individual’s thoughts or memories into a computer. However, these claims are greatly exaggerated and create unrealistic expectations for the capabilities of BCI technology. Currently, scientists have not yet developed a system with such capabilities, and current BCI technology lacks these functions (Portillo-Lara et al., 2021). It may continue to be a significant challenge to implement such a system in the foreseeable future. These types of claims are more commonly encountered in science fiction. While human imagination is limitless, it is important to acknowledge that not every imaginative concept is feasible in reality due to the inherent limitations and finite nature of human cognition and technological capabilities.

The human brain is an extraordinarily complex system, and the capabilities of current brain imaging technologies are limited. Furthermore, our understanding of the brain’s structure and functions remains quite basic. How is information, such as human memories, stored and transmitted within the central nervous system? What are the mechanisms underlying the generation of thoughts? How to accurately measure the information stored in central neurons and neural networks and convert it into digital signals that can be received by computers? What technologies would enable this conversion? These complex and difficult questions remain unanswered. The concept of using BCI to “download” thoughts and memories involves intricate cognitive processes and memory mechanisms within the brain. It requires a thorough understanding of how the brain encodes, stores, and retrieves information.

### 2.11 “Uploading” information into the brain using BCI

Some media outlets also claim that BCI technology has the potential to “upload” or “write” information directly into the brain. Similar to the exaggerated claims of “downloading” thoughts or memories, these assertions create unrealistic expectations for BCI technology and often resemble science fiction more than current technological capabilities.

How can information stored in computers be converted into a format that can be received and interpreted by central neurons or neural networks? Which technology could enable this conversion? These complex questions have yet to be answered. Moreover, the concept of “uploading” or “writing” information to the brain via BCI raises additional considerations about how the brain encodes, stores, and retrieves information.

### 2.12 “Significantly enhancing memory, cognitive, or behavioral performance” using BCI

Some researchers, developers, and manufacturers in the field of BCI, along with certain media outlets, claim that BCI technology, including neural stimulation and neurofeedback techniques, can significantly improve an individual’s memory, cognition, or behavioral performance. However, the reported efficacy of BCI may be limited to specific cases involving individuals with certain central nervous disorders. Furthermore, the degree of improvement varies, with some individuals experiencing only minor enhancements or none at all, emphasizing the need for accurate quantitative evaluation. Although there has been progress in this area, such reports tend to be inaccurate, overstating the current technological capabilities and possibly leading the public to overestimate the extraordinary efficacy of BCI. Research in this field is still in the exploratory phase and requires more thorough and in-depth investigation (Jamil et al., 2021).

### 2.13 BCI users are not part of the BCI system

**Figure 4:**
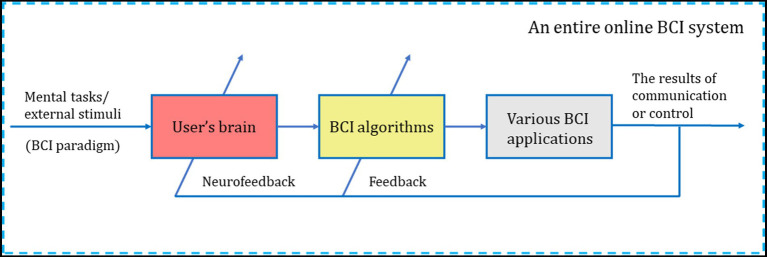
BCI users and their brains are key components of an entire online BCI system, which are directly connected to external devices. The BCI system is a typical human-in-the-loop (brain-in-the-loop) system. The arrows through the user and/or the BCI system indicate that they adapt to improve and maintain the correlation between the user’s intent and the BCI’s output (Krusienski et al., 2012; McFarland et al., 2012; Perdikis et al., 2018; Wolpaw et al., 2020).

Some literature and reports on BCI research tend to treat BCI users and the BCI system as separate, independent entities. However, it is crucial to acknowledge that BCI users and their brains are essential and integral components of the entire BCI system, directly interconnected with peripheral devices. The BCI system operates as a typical human-in-the-loop (brain-in-the-loop) system (Lyu et al., 2023), as illustrated in Figure 4. In the entire online BCI system, the user’s central nervous system serves as the signal source for interaction and control. The neural signals produced are the targets for decoding by BCI algorithms. Without the user, the BCI system becomes like “water without a source.”

As shown in Figure 4, successful online BCI operation requires effective interaction between two adaptive controllers (Taylor et al., 2002; Wolpaw et al., 2002, 2020; Krusienski et al., 2012; McFarland et al., 2012; Perdikis et al., 2018). One of these is the user’s brain, or the central nervous system (CNS), and the other is the BCI algorithm, which is responsible for processing and decoding brain signals. Therefore, users of BCIs are essential to the BCI system, as they engage in direct interaction with the BCI algorithm.

### 2.14 The ethics for BCI technology do not constitute a part of the BCI technology standards

Some BCI researchers, developers, and manufacturers tend to underestimate the importance of ethical issues, viewing the ethics of BCI technology as separate from its standardization. However, the development and potential applications of BCI technology have raised ethical concerns, making ethics an essential part of the standardization of BCI technology.

To date, there is relatively little literature on ethics within BCI research, and presentations on ethics at academic conferences focused on BCI are also limited. Additionally, these ethical discussions tend to attract fewer audience members. Further research into the ethical issues and guidelines for BCI technology is necessary, and these should be incorporated into the standards and regulations of BCI research and industrial practices. For example, the standardization of BCI technology systems should include ethical considerations for the medical application of implantable BCI technologies (Zhang et al., 2023), as illustrated in Table 1 (Zhang et al., 2023).

**Table 1:** Ethical considerations for medical applications of implantable BCI technologies.

Number	Ethical considerations
1	Ensuring patients’ right to informed consent when participating in research or using implantable BCI technologies
2	Reducing the risk of brain tissue damage caused by implantable BCI electrodes
3	Offering patients customized or personalized precision treatments with implantable BCIs
4	Reducing the risk of implantable BCIs impacting patients’ sense of identity or self-perception
5	Assisting patients with implantable BCIs in exercising and sharing their agency
6	Protecting the neural privacy of patients with implantable BCIs
7	Ensuring multidisciplinary collaboration in the clinical applications of implantable BCI
8	Adhering to the principle of benefit over harm and responsible use of implantable BCI
9	Ensuring patient access and ongoing usage of implantable BCI
10	Standardizing research reports and public understanding for implantable BCI
11	Requiring specific ethical considerations for certain diseases, disease-specific stages, or specific patients

### 2.15 Neural stimulation and neurofeedback technologies are not part of BCI category

Some researchers, developers, and manufacturers of BCI technologies, along with certain developers of neural stimulation and neurofeedback technologies (narrowly defined as neural modulation, as shown in Figure 2 of Section 2.7) do not acknowledge that these technologies are part of the BCI category. However, BCI includes non-invasive transcranial brain stimulation and deep brain stimulation (Conde et al., 2020), and neurofeedback is one of the earliest applications of BCI (Van Der Kolk et al., 2016).

Brain-computer interaction is typically defined as interactions primarily involving the output from the brain to external devices, while interactions involving input from external devices to the brain are referred to as narrowly defined neural modulation. However, these forms of neural modulation essentially involve interactions between the brain and machines (i.e., BCI), with neurofeedback creating a bidirectional closed-loop interaction.

### 2.16 Non-invasive BCI poses no ethical risk issues

Some BCI developers and manufacturers believe that non-invasive BCI poses no ethical risks, unlike invasive BCI. While non-invasive BCI does not directly damage brain tissue and does not have as significant ethical risks as invasive BCI, it may still have some irreversible effects on the user’s brain, such as impacts on neuroplasticity (Yuan et al., 2021). Are these effects beneficial or detrimental? If the use of non-invasive BCI causes irreversible negative changes in the user’s brain, it may consequently impact their physical and mental well-being (Zhang et al., 2023).

For non-invasive BCI, clinicians are responsible for customizing personalized BCI treatments for patients. This involves adapting to the neural diversity among individuals and the neural variability within an individual, as well as protecting patients’ neural privacy. Moreover, clinicians are tasked with assisting patients in making genuine life choices, and helping patients exercise and share their agency (Klein, 2020).

### 2.17 Neural privacy issues brought by BCI are severe

Some BCI developers, manufacturers, and media outlets, when reporting or discussing the ethical issues of BCI technology, believe that the neural privacy issues brought by BCI are severe. It remains unclear what specific personal privacy information, such as age, personality, hobbies, physical attributes, behavior, or personal experiences, might be encoded in the brain signals of BCI subjects or users. Although the central nervous data collected by BCI technology may contain private information of subjects or users, such as details about their health conditions, it is incumbent upon physicians to assist patients in establishing and managing areas of neural privacy concerning the information gathered and regulated by BCI devices (Klein, 2020).

Additionally, at the technical level, secure encryption algorithms can be used to encrypt related neural information, making it difficult to interpret privacy. At the legal level, actions involving the illegal collection of central nervous information through BCI are subject to legal actions based on privacy invasion laws and regulations (Zhang et al., 2023). Therefore, the neural privacy issues brought by BCI technology are not as severe as some imagine.

## 3 Discussion

Different people have different opinions on the current status and future of BCI, and every opinion will be subjected to the test of time and practice, continually refining existing viewpoints. Some views expressed in the review represent our current standpoint, which we will update as BCI technology evolves. We believe that BCI holds potential significant applications for specific individuals and look forward to its sustainable development.

The several inaccurate or erroneous conceptions and misleading propaganda about BCI, as elaborated on in the paper, may also receive different opinions from different individuals. Some issues require further discussion.

### 3.1 The limitations (shortcomings or weaknesses) and limits (maximum potential or boundaries) of BCI

It might be essential to acknowledge that every technology has its limitations and limits; they are not omnipotent, and BCI technology is no exception.

The limitations of BCI technology may be reflected in its dependency on the current levels of neuroscience and engineering technologies, which restrict its application scope and efficacy. For example, the user experience or satisfaction with current BCI systems is not very high, possibly leading to visual fatigue or mental workload for the subjects or users. Some BCI systems require subjects or users to undergo lengthy learning and adaptation, and deficiencies in decoding accuracy, stability, and response time may limit their efficacy or usability. Currently, the safety concerns of implantable BCI make subjects or users hesitant.

The limits of BCI may manifest in the theoretical and practical boundaries of the technology itself. Despite ongoing research and technological advances, BCI still faces significant challenges in decoding complex brain activities and intentions and achieving highly personalized interactions.

### 3.2 What efficacies can the current BCIs provide? What level of performance have they achieved? To what extent have they been developed? What can they not do?

Currently, experimental research suggests that the potential efficacies of BCI include monitoring (using BCI systems to monitor and assess an individual’s brain state); replacement (the output of BCI systems can substitute for natural outputs lost due to injury or disease); improvement/restoration (primarily aimed at the rehabilitation field, to improve symptoms of a disease or restore certain functions); enhancement (mainly targeting healthy individuals, to achieve improved and expanded capabilities); and supplementation (primarily targeting the control domain, adding brain-controlled methods as a supplement to traditional single control methods, achieving multimodal control).

Presently, BCI is mainly in the demonstration stage in laboratories or under clinical research for validation and testing, showing certain efficacies for specific individuals. However, the usability, efficacy, user satisfaction, and usage of BCI systems need substantial improvement.

Under the current conditions of technology, knowledge, and resources, as mentioned earlier, BCI cannot identify an individual’s arbitrary intentions or thoughts but can only classify a pre-designed set of mental tasks or specified set of external stimuli with certain accuracy; it is not possible for BCI to “download” a person’s thoughts or memories into a computer; nor is it feasible for BCI to “upload” or “write in” intentions or information to the brain.

### 3.3 What is the current status of the industrial translation of BCIs? What are the pathways for BCIs to be translated into practical applications?

Currently, there are relatively few BCI systems that are significantly effective, safe, reliable, and approved for market sales. The few BCI systems that have been approved for market are primarily designed for patients with specific diseases. There are fewer BCI products available for healthy individuals in specific scenarios, and their functionalities are relatively limited. Most BCI research and development is still in the clinical phase, which entails lengthy clinical trials. At present, the sales are mainly for BCI products used in scientific research, and the approval process for BCI medical devices is exceptionally long, particularly for innovative technologies. As previously mentioned, since the BCI field is in its infancy, the market size is unknown, which places high commercial risks with potential manufacturers (Ramsey, 2020).

The most anticipated applications of BCI are likely to be in medical clinical settings first. It is recommended to accurately identify and focus on the needs of patients with specific diseases, that is, to determine the optimal or most suitable application scenarios for BCI, and to carry out customized personalized design, verify and significantly enhance efficacy, with the aim of achieving industrial translation. It is difficult for BCI to be effective or provide rehabilitative benefits for many diseases, but BCI can target a few specific “most suitable or applicable disorders,” such as focusing on the motor function rehabilitation of patients with stroke and spinal cord injuries.

### 3.4 Regarding the potential applications of BCI, which could be realized in the near term, which within the next 5 years, which within the next 10 years, and which are still dream or imagined situations?

Regarding this issue, different people have different views or predictions. In the near future, practical applications of BCI systems may include non-invasive BCI devices for upper or lower limb rehabilitation, aimed at helping patients, such as those who have suffered strokes, to restore the function of their arms or legs. These applications also extend to simple entertainment and gaming uses, as well as brain state monitoring based on BCI. Within the next 5 years, more practical, complex BCI control devices may emerge, such as BCI-controlled prosthetics with fine control, along with more advanced neural rehabilitation technologies based on BCI. Within the next 10 years, we might see more sophisticated BCI applications, such as advanced neural enhancement devices. Some applications considered “dreams,” such as direct brain-to-brain communication, may take even longer to realize, if they are fundamentally possible at all.

### 3.5 Is it necessary to conduct “research on countering BCI systems”?

“Research on countering BCI systems” aims to interfere with, attack, or undermine the security and stability of BCI systems, seeking methods or strategies to counter BCI systems. This may include using unethical means to affect their performance or destroy their functionality. In this context, “countering” refers to actions or behaviors specifically targeting the BCI systems. In contrast, research on BCI systems focuses on enhancing their stability, reliability, and robustness, which is a current challenge in the BCI research community. It includes how BCI systems handle adversarial signals, noise, or attacks, and explores the performance of BCI systems in adversarial environments or conditions.

The purpose of BCI is to improve the quality of life or work efficiency for patients, disabled individuals, or healthy individuals. Some believe that BCI technology is still at an early stage of development, facing significant challenges. The breakthrough in BCI technology requires the joint efforts of multidisciplinary scientists (Bergeron et al., 2023) to narrow the gap between research and practical application (Allison et al., 2012). Currently, BCI systems are relatively fragile, and it is relatively easy to disrupt or counter these fragile systems. For example, in EEG-based BCI systems, obstacles in EEG signal processing include a low signal-to-noise ratio, limited spatial resolution, and the presence of strong artifacts, such as those caused by eye movements, line noise, and cable movements. These factors result in the lower stability, accuracy, or reliability of such systems. However, some argue that research on countering BCI systems is necessary.

### 3.6 Suggestions for reducing inaccurate or erroneous conceptions and misleading propaganda about BCI

The public’s inaccurate or erroneous conceptions of BCI technology are related to misleading hype by certain BCI researchers, manufacturers, regulators, and media outlets. To avoid or stop the exaggeration or hype of BCI’s efficacy, it is necessary to increase public education on BCI, accurately introducing the basic knowledge, current status, and potential of BCI technology. Evaluation standards for BCI research and products, such as efficacy, usability, user satisfaction, and usage, should be established, and it is crucial to ensure that the disseminated information is objective. Media and BCI-related enterprises should be encouraged to engage in responsible reporting and promotion, avoiding misleading the public or consumers for the sake of profit.

#### 3.6.1 Popularizing scientific knowledge of BCI to the public

Currently, there is a gap between the public’s high expectations and the actual technological capabilities of BCI. To narrow the gap, it is important to enhance the public’s scientific understanding of BCI technology. This can be achieved by conducting accurate and scientific public education activities about BCI, which would help reduce misconceptions and overly optimistic expectations. It is suggested to showcase actual case studies and limitations of BCI technology to encourage fact-based understanding and discussion.

It is important to provide the public with accurate popular science knowledge about BCI. This can be achieved through publishing BCI popular science books, organizing popularization activities such as public courses, online educational platforms, or hosting lectures by BCI experts. These initiatives aim to inform the public about the basic principles, current progress, and potential applications of BCI technology. When popularizing BCI to the public, it is essential to avoid unscientific, biased, misleading, or sensationalized communication.

#### 3.6.2 The responsibilities of BCI researchers and developers

BCI researchers and developers ought to have a profound understanding of BCI technology, and rationally evaluating its level of maturity as well as its practical limitations. It’s crucial for them to objectively assess the current development level, functionalities achieved, and limitations of BCI technology. Additionally, they need to recognize the discrepancy between the maturity of BCI technology and the general public’s perception, which is usually inaccurate or erroneous.

#### 3.6.3 The responsibilities of BCI manufacturers

BCI manufacturers, during the financing phase and while pursuing profits, should avoid engaging in hype, false advertising, or misleading propaganda about BCI. It’s imperative that they should also avoid using unfair means, distributing counterfeit or inferior BCI products, and infringing on consumer rights. Moreover, when marketing BCI products, it is essential to provide detailed guidance and training to users, ensuring they correctly understand and use BCI technology.

#### 3.6.4 The responsibilities of regulators of BCI technology

Some regulators in the field of BCI technology should enhance their professional expertise in BCI. It is crucial for them to evaluate the technology’s maturity, practical limitations, and prospects for industrial translation in a scientific, objective, and rational manner. Additionally, they should avoid inaccurate or erroneous conceptions and misleading propaganda about BCI.

#### 3.6.5 The responsibility of the media in reporting BCI

When reporting on BCI technology, the media should avoid being manipulated by some BCI researchers, developers, manufacturers, or regulators of BCI technology, and avoid providing inaccurate information or engaging in hype. To ensure accuracy and scientific integrity, BCI-related content in media reports should undergo scrutiny by experts who possess rigorous scientific knowledge in the field of BCI.

By implementing these suggestions, the goal is to diminish public misunderstandings about BCI technology, thereby enhancing their accurate comprehension of this advanced field and fostering its responsible development. Moreover, interdisciplinary collaboration can bring together knowledge and resources from various fields to jointly address key scientific and engineering challenges in the development of BCI technology. Furthermore, strengthening cooperation with fields such as ethics, law, and sociology is essential to ensure that the development of BCI technology is both scientific and compliant with ethical and legal requirements.

## 4 Conclusion

The article focuses on addressing inaccurate or erroneous conceptions about BCI prevalent among certain segments of the public, as well as misleading or overhyped publicity in some media outlets, and even among BCI researchers, developers, manufacturers, and regulators. It elaborates on several inaccurate or erroneous conceptions and misleading propaganda about BCI, and provides suggestions to reduce such inaccuracies, misconceptions, and misleading propaganda about BCI.

## Author contributions

YC: Investigation, Writing – original draft. FW: Writing – review & editing. TL: Writing – review & editing. LZ: Writing – review & editing. AG: Writing – review & editing. WN: Writing – review & editing. PD: Conceptualization, Investigation, Writing – review & editing. YF: Conceptualization, Funding acquisition, Investigation, Project administration, Supervision, Writing – review & editing.
